# Microbial technologies as an ecological tool for advancing environmental sustainability

**DOI:** 10.3389/fmicb.2026.1875044

**Published:** 2026-07-01

**Authors:** Aminat Oyiza Musa, J. M. I. Yarboe, D. A. Undie, O. O. Akinpelu, H. S. Samuel, E. E. Etim

**Affiliations:** 1 Department of Microbiology, Bayero University, Kano, Kano State, Nigeria; 2 ChemBio Nexus Research Group, Abuja, Nigeria; 3 Department of Chemistry, Faculty of Science, Islamic University of Madinah, Madinah, Saudi Arabia; 4 Department of Chemical Sciences, Federal University Wukari, Wukari, Taraba, Nigeria; 5 Cerba Lancet Laboratory, Ibadan, Oyo, Nigeria; 6 Department of Biochemistry, Federal University of Technology, Ikot Abasi, Nigeria

**Keywords:** environment, environmental sustainability, industrialisation, microbial technologies, microorganisms, pollutants

## Abstract

The global drive for environmental sustainability demands accelerating efforts in searching for sustainable, innovative solutions to address the environmental consequences of industrial operations. While these operations are driven by the increased demand to satisfy human needs, they have also imposed risks of natural resources scarcity and compromised planetary health. However, sustainable microbiology has emerged as a popular scientific discipline capable of delivering innovative sustainable microbial technologies for addressing these challenges. Microbes, such as *Pseudomonas* and *Bacillus* species, have useful applications in bioremediation. Many microorganisms can produce sustainably relevant industrial products, such as biopesticides, bioplastics, biofuels, and biomaterials. For example, *Cupriavidus necator*, *Escherichia coli*, and *Bacillus* species can synthesise biopolymers that can be used as precursors for manufacturing bioplastics, microalgal systems can serve as biofactories for biofuel synthesis, whereas *Synechocystic* sp. and microalgae can assist with reducing carbon footprints through biological carbon capture and utilisation. Through biotechnological approaches, such as genetic engineering, synthetic biotechnologies, and omics, microbial functions can be enhanced and optimised for specific environmental issues, considerably boosting process efficiency and reducing production costs. This study explores the environmental liabilities and evaluates the potential of microbial technologies as innovative strategies for their smooth transition into sustainable and eco-friendly industrial operations. It provides insights into biotechnologically enhanced microorganisms and microbial processes as ecological transformative tools for achieving environmental sustainability. It further highlights scalability, safety, ethical, and social acceptance as crucial concerns while emphasising the need for further investigation into immediate and long-term environmental impacts of the technology.

## 1 Introduction

Globally, for several years, the environment has continued to face threats, such as environmental degradation and greenhouse gas emissions. The world’s shift towards industrialisation remains a key factor driving these issues (Bala et al., 2022) through human activities. Considering this evidence, these challenges may have arisen from the excessive utilisation of the Earth’s natural resources to produce various products for human consumption. While these resources are critical to the survival of all living organisms, such as plants, animals, and humans (Akinsemolu, 2023), utilising them sustainably is essential for both the continuation of life and the planet’s health. Intensive research into challenges driving an unsustainable environment reveals various human practises and pollutants damaging the environment (Bala et al., 2022; Omotehinse and De Tomi, 2023; Punia and Singh, 2025), such as waste materials from steel, battery, and mining industries (Bala et al., 2022). These industries are involved in a series of routine activities and processes, ranging from metal extractions and synthesising chemicals to utilising non-renewable resources for energy supply. This often results in the deposition of chemical wastes that can be carried by runoffs into groundwater or freshwater bodies, making them unsafe (Bala et al., 2022). Of importance, mining operations often result in environmental accumulation of toxic heavy metals and soil destruction (Omotehinse and De Tomi, 2023), which releases stored carbon stocks in the soil, and along with fuel combustion, they contribute to the growing climate change issue (Punia and Singh, 2025). This industry can be said to be of ecological interest because it provides the starting raw materials to the steel and battery industries, indirectly accounting for their considerable negative environmental inputs. Similarly, the petroleum industry generates considerable forms of environmental pollutants, including oil sludge, greenhouse gases, oil spills, and effluents and waste discharge, which affect the natural physical and biological systems in the environment, such as humans, plants, animals, climate, and infrastructures (Ogolo et al., 2022). Furthermore, the persistence of heavy metals in the environment, including the aquatic environment, is attributed to their low degradation power, and combined with their hazardous nature, they are considered harmful environmental pollutants (Zaynab et al., 2022). However, in agriculture, where pesticides are increasingly important for pest control, their widespread use has adversely impacted the environment too (Mali et al., 2023). Pesticides, which are formulations that constitute majorly harmful chemicals, often leave traces of toxic chemical residues in locations where they are used, degrading such environments. Furthermore, plastic waste is another critical environmental issue of global concern. They accumulate in different environmental compartments, such as terrestrial and aquatic environments, and can poorly decompose to generate macro- and microplastics (de Souza and Gupta, 2024), which in turn introduce various toxic contaminants into the environment owing to their appreciable adsorption property (Kinigopoulou et al., 2022). This attribute could compound the environmental burden driven by plastic waste. Collectively, these environmental issues are serious global issues, making prioritising the environment and advancing its sustainability an important global goal (Akinsemolu, 2023). Although strategies such as renewable energy technology and strict emissions standards are demonstrated to have potential for achieving environmental sustainability (Khanam et al., 2023), sustainable microbiology could create innovative microbial solutions to a wide array of environmental issues and lead the world to a sustainable future, as shown by studies from Verstraete et al. (2022) that established that microbial technologies can deliver sustainable mitigation strategies against climate change long-term effects. Sustainable microbiology is an emerging sub-discipline concerned with using microorganisms or microbial technologies to create sustainable solutions for advancing sustainability (Akinsemolu, 2023). Obviously, the viability of this approach is based on the diverse metabolic capabilities of microorganisms and their ability to quickly respond and adjust to changes in the environment. Several studies have examined utilising microbial solutions for the removal of various environmental pollutants. Zeenat et al. (2021) discussed the biodegradation of plastics by microorganisms, proposing their potential in the sustainable removal of harmful plastics from the environment, while de Souza and Gupta (2024) reported that the microbial synthesis of biopolymers using biowaste as a substrate could serve as an alternative to petrochemical-based polymers. These innovative strategies successfully present dual environmental benefits: replacing petrochemical-based plastics and removing organic waste from the environment. Furthermore, Faulkner et al. (2024) investigated the ability of photosynthetic bacteria to metabolise carbon dioxide to produce biomaterial precursors for synthesising bioplastics. This not only adds to the existing methods for bioplastic production but also helps to tackle climate change through the removal of carbon from the atmosphere. But none of these studies addressed environmental issues in the context of advancing environmental sustainability. Similarly, other available knowledge of harnessing microbial technologies for addressing environmental issues is in the areas of health (Das et al., 2023), ecosystem services (Oro et al., 2024), and clean water (Saravanan et al., 2023). Thus, this article aims to explore the knowledge of using microorganisms and the potential of biotechnologically enhanced microorganisms for advancing environmental sustainability. It synthesises a wide range of publications related to environmental implications and climate change impact of industrial activities, as well as provides comprehensive coverage on the potential of sustainable microbiology to offer sustainable and environmentally sound alternative approaches to transform industrial processes towards ensuring environmental sustainability.

## 2 Microbial technologies in achieving environmental sustainability

Eco-friendly and cost-effective alternatives have been explored to protect and preserve the environment in recent times. Amongst these, bioremediation, an advanced and environmentally sustainable technology, has been established as a breakthrough process for treating contaminated environments (Verstraete et al., 2022; Pereira et al., 2019; Wyckhuys et al., 2019). Bioremediation offers a natural solution that utilises biological microorganisms to assimilate, digest, degrade, transform, or immobilise hazardous pollutants into less toxic forms. Through processes such as biodegradation, bioaccumulation, bioaugmentation, biostimulation, biotransformation, and microbial consortia-based systems, bioremediation effectively reduces the toxicity of industrial effluents, heavy metals, xenobiotic compounds, pesticides, and chlorinated pollutants (Chinthala, 2025; Joutey et al., 2013; Singh et al., 2008). While some fungi are now used for the remediation of heavy metals like lead and arsenic, bacterial species like Bacillus and Pseudomonas have been widely used in the breakdown of hydrocarbons (Ayilara and Babalola, 2023). Molecular oxygen is introduced into hydrophobic aromatic rings by bacterial monooxygenases and dioxygenases during aerobic hydrocarbon pathways. This process breaks the stability of the ring, transforming them into intermediates like catechol, which the bacteria can then easily cleave and channel directly into the microbial tricarboxylic acid (TCA) cycle for energy. Bioremediation can be carried out using either ex situ (excavating contaminated materials for treatment elsewhere) or *in situ* (treating toxins directly at the site) methods, depending on the remediation goal and environmental matrix (Hlihor and Cozma, 2023). Notably, Akhtar and Mannan thoroughly examined mycoremediation techniques for eliminating environmental contaminants from agricultural practises (such as pesticides, herbicides, and cyanotoxins) and industrial sources (such as heavy metals and aromatic hydrocarbons), emphasising their potential to address the expanding global pollution problem (Akhtar and Mannan, 2020). Furthermore, the effectiveness of bioremediation can be improved by bioaugmentation, which introduces specific microbial strains to increase pollutant breakdown efficiency, or biostimulation, which provides nutrients or oxygen to boost native microbial communities (Singh et al., 2008; Ayilara and Babalola, 2023; Hlihor and Cozma, 2023). Microbial activities such as biosorption, bioaccumulation, and metal reduction limit the environmental bioavailability. When toxic metals are in contact with other substances, the valence state of them is altered by certain microbial reductases during the process. For example, they change the highly soluble, carcinogenic Hexavalent Chromium [Cr(VI)] into a safer, stable, insoluble Trivalent Chromium [Cr (III)]; and anaerobic sulphate-reducing bacteria produce hydrogen sulphide (H2S) gas which binds to chemically precipitate dissolved metals into harmless stable metal sulphides (Ayilara and Babalola, 2023; Sun et al., 2024; Narayanan et al., 2023). Biodegradation is used as a focused technique in designed remediation systems to break down organic pollutants into less hazardous and simpler products. Microbial enzymes, such as oxygenases, peroxidases, dehydrogenases, and hydrolases, induce biodegradation in both aerobic and anaerobic settings by starting the conversion of complicated contaminants into less hazardous intermediates (Narayanan et al., 2023).

**Figure 1:**
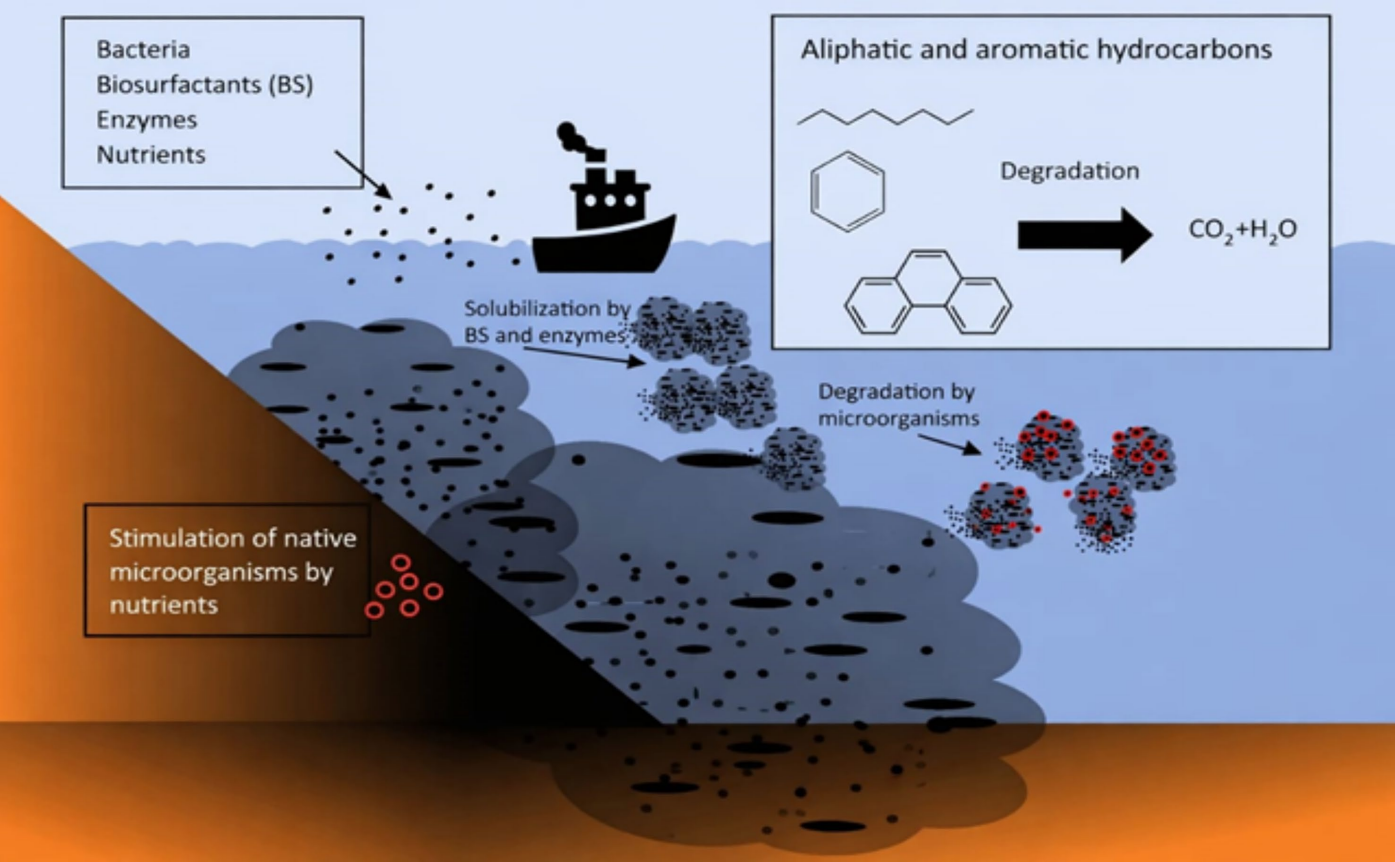
Microbial mechanisms of bioremediation of oil spills (Tedesco et al., 2024). Source: https://creativecommons.org/licenses/by-nc-nd/4.0/.

In aerobic environments, organic pollutants are usually oxidised by microorganisms using oxygen as the terminal electron acceptor, resulting in the production of carbon dioxide and water (Pereira et al., 2019; Joutey et al., 2013). Anaerobic biodegradation uses nitrate, sulphate, iron, or carbon dioxide as electron acceptors in alternate routes; this is especially important in groundwater systems and oxygen-limited sediments. Reductive dehalogenase enzymes are employed by anaerobic bacteria in organohalide respiration to swap chlorine atoms out of chlorinated solvents like trichloroethylene (TCE), resulting in the conversion of non-toxic ethylene to chlorine in a specific sequence (Joutey et al., 2013; Narayanan et al., 2023). According to Sun et al.’s (2024) study, microbial consortia, sulphate-reducing bacteria, and extremophilic bacteria have demonstrated significant promise for heavy metal remediation, especially under challenging circumstances, such as high salinity, low pH, and higher metal concentrations. Through enzymatic hydrolysis, oxidation, and reduction pathways, functional bacteria and fungi that were isolated from polluted matrices have demonstrated the capacity to break down pesticide compounds into less hazardous intermediates, A clear instance of this is seen when microbial organophosphate hydrolases (OPH) specifically target and break the characteristic P–O or P–S ester bonds found in complex organophosphate pesticides, rendering them non-toxic before cellular uptake (Joutey et al., 2013; Ahmad et al., 2021). In well-oxygenated environments, microorganisms perform more effectively because increased oxygen availability boosts aerobic metabolic activity and speeds up biodegradation processes (Joutey et al., 2013). Microbial enzymes are essential for biotransforming complex pollutants into less harmful and usable forms as shown in Figure 1 (Tedesco et al., 2024), which summarises the microbial metabolic pathway involved in bioremediation of oil spills.

### 2.1 Microbial bioproducts as a future sustainability catalyst

#### 2.1.1 Bioplastics in the management of waste

The primary environmental benefit of microbially degradable bioplastics is their biodegradability, or the capacity of microorganisms to naturally break them down. Like most plastics, microplastics have a hundreds-year lifespan. However, in a matter of years, microorganisms in the environment can totally decompose polyhydroxyalkanoates (PHA) and other microbial polyesters into carbon dioxide and water (Jem et al., 2010; Badji et al., 2018).

The quantity of persistent plastic waste that finds its way into terrestrial and marine settings is probably going to be significantly reduced by this feature. Furthermore, because the carbon in these biopolymers comes only from biogenic sources, which are more sustainable and environmentally friendly, life cycle assessments (LCA) have demonstrated that their greenhouse gas emissions are significantly lower than those of conventional equivalent fossil fuel-based materials (Felipe de Lima Andreata et al., 2025; de Mello et al., 2025). The application of PHA and other bioplastic products was proved to reduce the emission of greenhouse gases through several LCAs conducted by specialists. However, this effect is greatly dependent on the process. Biomass treatment and pretreatment have been considered as the most energy-consuming steps and the drivers of various environmental impacts of PLA and PHB from lignocellulosic waste (Senila et al., 2024). While comparing PHA production from CO₂ and glucose from corn, lower environmental impacts for the first process were detected for all impact categories except chloroform extraction, which turned out to be a significant issue for improvement (Koch et al., 2023). The new approach to an LCA includes the category called “climate tipping,” where PHA-based films showed better results; nevertheless, it should be kept in mind that PHA-based packaging can have greater environmental impacts than PE and PP packages when yield is low and molasses is used optimally (Vea et al., 2021). Recent advances in synthetic biology and genetic engineering have made it possible to modify these microbial strains to consume inexpensive substrates. Like molasses and agricultural waste, thus separating the manufacture of plastic from fossil fuels.

For instance, microorganisms like *Cupriavidus necator*, *Escherichia coli*, and *Bacillus* species have been used to alter some inexpensive agricultural waste and molasses by introducing genes that produce PHAs, eliminating carbon-wasting reaction pathways, and increasing the enzyme’s efficiency, which lowers production costs and minimises waste pollution (Jem et al., 2010; Le et al., 2025). This is accomplished by decomposing these agricultural wastes, such as cassava peels, corn waste, sugarcane bagasse, etc., and letting them go through fermentation. During this process, these bacteria eat the wastes and turn the sugars into biodegradable plastics that are stored inside their cells. These PHAs can then be extracted and processed into products that are useful (Le et al., 2025; Jafari and Hejazi, 2024; Mijatović et al., 2018).

**Figure 2:**
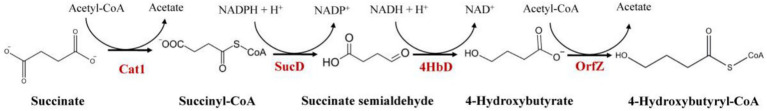
4HB-CoA production pathway in *Clostridium kluyveri* (Mitra et al., 2021).

Scientists and engineers use bacteria and yeast to improve metabolic pathways, which enables efficient production of plastics from renewable feedstocks. Biopolymers such as PHAs are produced by genetically modified organisms (GMOs) and are subject to biotechnology laws. The U. S. has several government agencies regulate such GMO products. Yet, there are limitations on how current laws can regulate advancements in genome editing technology, especially when used for the creation of bioplastics (Shams et al., 2024). Nevertheless, it is worth mentioning that safety assessments emphasise that the release of or even improper containment of GEMs will inevitably lead to their dissemination and gene swapping with the natural microorganisms and persistence in the environment (Chemla et al., 2025). Therefore, appropriate risk assessment before the release of the product and proper monitoring are needed. These innovations reduce costs, cut fossil fuel use, and support environmentally sustainable plastic manufacturing. This can easily be done by using bacteria, which are able to produce a great variety of polymers like polyesters, polysaccharides, polyamides, and, in some cases, even polyphosphates from corresponding monomers. This synthesis requires the two key enzymes, propionyl-CoA transferase, whose production pathway is shown in Figure 2, and PHA synthase. The enzymatic activity of propionyl-CoA transferase converts lactic acid into lactyl-CoA. Lactyl-CoA will then be polymerised by the PHA synthase. Another type of polymer that bacteria can produce includes dextran, xanthan, and polyhydroxyalkanoates (PHAs), which include a large group of polymers such as polyhydroxybutyrate (PHB), polyhydroxyvalerate (PHV), and other polyhydroxyalkanoates (PHAs). Some examples of bioplastics which are obtained from the metabolic process of bacteria include polyhydroxyalkanoates (PHAs), which is a group of polymers that contain polyhydroxybutyrate (PHB), polyhydroxyvalerate (PHV), polyhydroxyhexanoate (PHH), and poly(3-hydroxybutyrate-*co*-3-hydroxyhexanoate) (PHBH) (de Souza and Gupta, 2024; Notonier et al., 2021; Mitra et al., 2021; Rehm, 2010).

Recent work shows that PHA is technically feasible at industrial scale, but still expensive. A simulation of a full-scale plant using cheese whey by products and the halophilic archaeon *Haloferax mediterranei* produced about 9,700 t/year of PHBV with 87% process efficiency; the breakeven price could be brought down below about 4 US$/kg when enzymes and media are recycled, but economics are very sensitive to feedstock price (Wang et al., 2022). The current minimum selling prices of the PHA are typically 4–8 US$/kg, depending on substrates, fermentation and downstream extraction (Fadipe et al., 2026). Some of the things that make PHA cost competitive and scalable are novel approaches which include the use of mixed microbial cultures, waste feedstock and improved recovery (Khatami et al., 2021; Bolla et al., 2025). Although PHA can already be produced in pilot and commercial plants, high production cost and process complexity are still the main industrial barriers.

#### 2.1.2 Biofuels

There is also a microbial revolution unfolding in the energy sector. The third- and fourth-generation biofuels are under development from microalgae and genetically modified Cyanobacteria that can convert sunlight and CO₂ into lipids to produce ethanol, which can be used as fuel. Moreover, anaerobic digestion processes using methanogenic archaea have become popular for the degradation of organic waste from municipalities to biogas. These biotechnological aspects not only generate renewable energy but also reduce pressure on natural resources by giving value to the waste feedstock that would contaminate landfills, making them useful, which encourages recycling. Microbial enzymes are used by the biofuel sector to transform biomass into sustainable energy sources. These enzymes are primarily sourced from fungi (e.g., *Trichoderma reesei and Aspergillus niger*) and bacteria (e.g., *Clostridium thermocellum*) (Jalandra et al., 2025). *Trichoderma reesei* uses submerged fermentation to produce cellulases. Cellulose is broken down into sugars that can be fermented. Plant biomass’s hemicellulose components are broken down by *Aspergillus niger* hemicellulases, making the manufacture of biofuel more efficient. Transesterification uses *Candida rugosa* lipases to convert oils and fats into biodiesel (Arshad et al., 2025).

#### 2.1.3 Biopesticides

The application of microbes in agriculture has been recognised as a very good alternative to the application of chemical compounds, which persist in the environment for a long period of time. Microbial biopesticides use bacteria such as *Bacillus thuringiensis*, fungi such as *Trichoderma,* and viruses to control the activities of pests and reduce their populations. Recent advances have been made by creating microbial consortia, which are combinations of different microbial species which can be used synergistically to boost the tolerance of plants and control diseases more effectively. This approach to biopesticides minimises the amount of chemicals used on arable land and helps to overcome the problem of pest resistance to chemical compounds. Biotechnology has been able to apply microbes as efficient biofactories to produce biopesticides that are safer and more environmentally friendly than synthetic agrochemicals, which remain in the environment for a long period of time. Microorganisms such as *Bacillus*, *Pseudomonas, Streptomyces*, *Trichoderma,* and entomopathogenic fungi are commonly used because they produce bioactive secondary metabolites, which exhibit insecticidal, fungicidal, and bactericidal activities naturally. Microorganisms can be manipulated through controlled fermentation processes to produce insect toxins, enzymes, lipopeptides, antibiotics, and volatile organic compounds, which affect the physiology of pests, inhibit their growth, and enhance defence mechanisms in plants to withstand the attack of these pests. For example, *Bacillus thuringiensis* is commonly used as a bioinsecticide. This bacterium produces insecticidal crystal proteins, which are known as “Cry proteins”, which affects the larvae of certain insects. Microbial bioactive metabolites can also be blended with plant-derived phytochemicals to produce synergistic biopesticides. This indicates that microbial biotechnologies can produce environmentally friendly biopesticides that support integrated pest management for sustainable agriculture development (Seenivasagan and Babalola, 2021; Verma et al., 2024; Rezaee Danesh et al., 2025).

#### 2.1.4 Biomaterials

**Figure 3:**
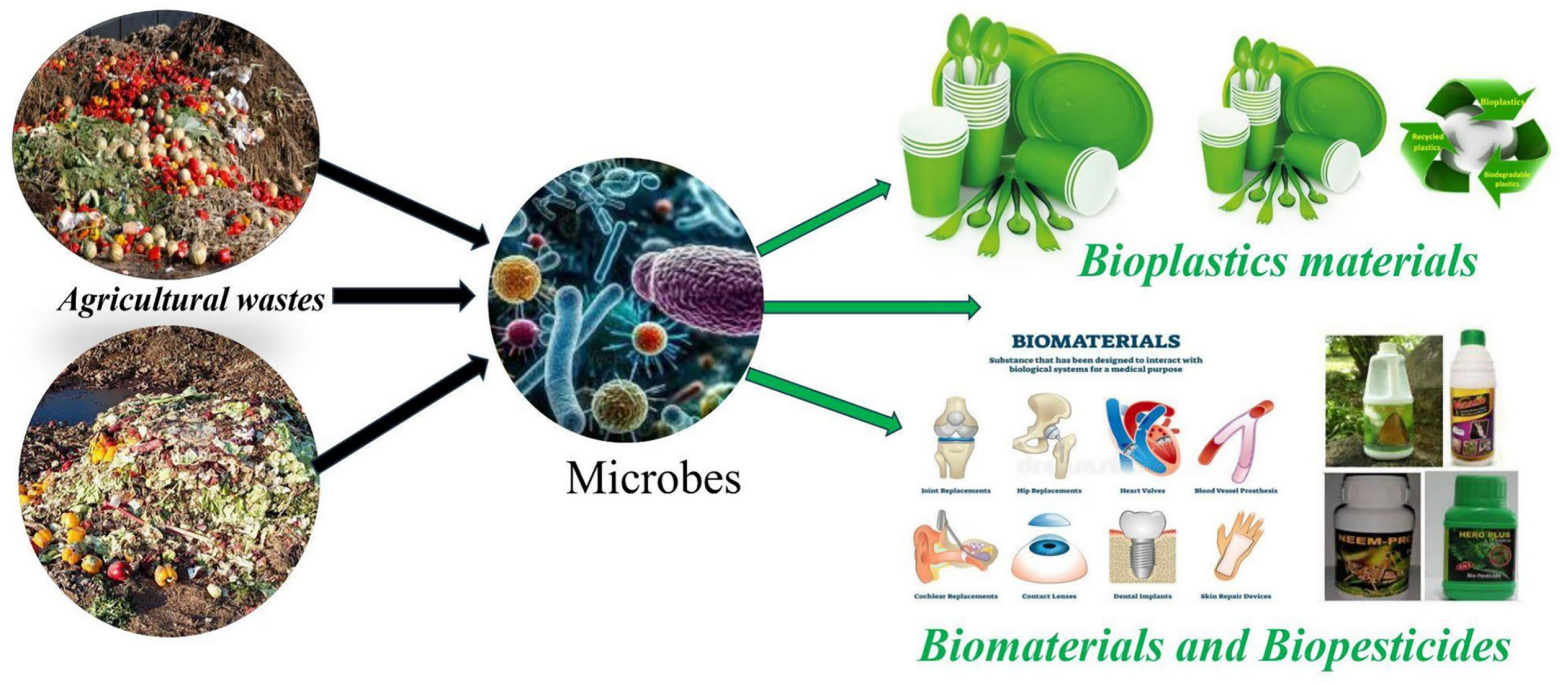
Schematic illustration of the role of microbes in transforming agricultural waste into sustainable products such as bioplastics and bio-based materials (Biswal et al., 2020).

Biomaterials are materials that can be used safely to interact with living tissue and are used for different purposes, including dental implants, heart valves, artificial skin, pacemakers, and tissue repair. Biomaterials are very significant in the replacement and repair of damaged organs and the improvement of the quality of life. These biomaterials have significant advantages that include the ability to be produced from renewable resources, such as agricultural wastes, and to be biodegradable/recyclable, as seen in Figure 3, which includes bioplastics, biofuels, and biopesticides, amongst others. They also contribute to the development of green technologies that reduce waste generated by non-biodegradable materials and the practise of “eco-friendly” manufacturing methods that ensure the protection of human health and the future of our environment (Biswal et al., 2020).

### 2.2 Microbial technologies as a complementary strategy for climate change mitigation

Biological processes, including bio-carbon sequestration, bioenergy production, and soil carbon improvement, driven by microorganisms such as bacteria, algae, and fungi, play a critical role in capturing atmospheric CO₂ and turning it into stable organic or inorganic forms, a process known as microbial bio-carbon sequestration. Microbes regulate major carbon flows through the fixation, transformation, and breakdown of organic matter. Through the conversion of aqueous organic carbon into refractory forms, ocean bacteria and archaea lower atmospheric CO₂ concentrations. Long-term carbon storage and climate management are greatly aided by this mechanism, which efficiently stores carbon in the deep ocean for hundreds to millennia (Zahed et al., 2021; Zhu et al., 2022). Using sunshine, photosynthetic organisms transform CO₂ into biomass, which can then be further processed to create biofuels or biofertilisers. Through acetogenesis, some bacteria, such as *Acetobacterium woodii*, use CO₂ as a carbon source to produce industrially useful acetic acid (Zhu et al., 2022). A carbon-rich substance made from organic waste; biochar increases soil microbial activity and encourages carbon storage. Applying biochar to agricultural regions increases soil fertility and sequesters carbon for decades by providing a home for carbon-fixing microorganisms. By working together, biochar and microorganisms can further improve environmental sustainability by lowering the production of greenhouse gases like nitrous oxide and methane (Zaidi et al., 2022). Through several interrelated processes, the synergistic interaction between soil microbes and biochar improves carbon stabilisation and reduces greenhouse gas emissions. By physically shielding organic matter and encouraging its adsorption, biochar’s porous shape and chemically resistant carbon matrix slow down microbial mineralisation and increase the soil’s long-term carbon persistence. Increased carbon incorporation into stable microbial biomass and necromass results from concurrent improvements in soil aeration, moisture control, and pH buffering. These physicochemical changes restrict anaerobic microsites that favour methanogenic archaea, hence lowering methane (CH₄) production while encouraging methane oxidation by methanotrophic bacteria. Furthermore, by adsorbing ammonium and nitrate, biochar increases nitrogen retention and decreases excess nitrogen availability, which fuels the production of nitrous oxide (N₂O). Improving redox balance and encouraging complete denitrification to dinitrogen (N₂), the biochar–microbe interaction reduces N₂O emissions. Together, these strategies promote carbon sequestration and lower greenhouse gas fluxes by rerouting soil biogeochemical pathways (Zhang et al., 2021). Microbial electrosynthesis is a new field that uses electroactive microorganisms to absorb CO₂ and transform it into useful chemical molecules. These bacteria, like *Geobacter sulfurreducens,* power CO₂ reduction processes using electricity. In addition to reducing carbon emissions, this technique promises industrial uses such as the manufacturing of biofuels, polymers, and pharmaceuticals (Kumar et al., 2025).

Reducing the potent greenhouse gas methane requires methanotrophic bacteria. These microbes transform methane into less hazardous compounds like CO₂, which can then be captured and stored. On the other hand, controlled methane production in biogas facilities where the gas is trapped and utilised as renewable energy is being studied using methanogenic archaea. In anaerobic conditions, these organisms generate methane (Onyeaka and Ekwebelem, 2023). Microbial populations in the soil are crucial to the carbon cycle. For instance, mycorrhizal fungi boost soil organic carbon storage by forming symbiotic interactions with plant roots. Similarly, decomposer bacteria and fungus turn plant waste into humus, a stable form of carbon. Agricultural practises that promote microbial diversity, like reduced tillage and organic farming, can significantly boost soil carbon stocks (Kumar et al., 2018; Lu et al., 2015; Ravichandran et al., 2024).

#### 2.2.1 Industrial applications

Microbial biotechnology has transformed industrial processes in several industries by utilising microbes’ special capacity to create useful molecules, break down contaminants, and improve efficiency. Significant developments in this area in recent years have increased the range and effectiveness of industrial applications, offering long-term answers to pressing global issues. A few potential applications of microbial technologies in industries are examined in this article (Osman, 2024). With the use of genetic engineering and synthetic biology, scientists can genetically alter microbes to improve their potential for use in industrial settings. Engineers can now create unique metabolic pathways and carry out genomic modifications in bacteria alongside optimising substrates to effectively manufacture various valuable compounds. Precision engineering has decreased reliance on conventional chemical synthesis techniques and expedited the development of ecologically friendly procedures. The primary objective of microbial metabolic engineering is to optimise their metabolism to boost production and productivity. By reducing production costs and boosting efficiency, this tactic increases the profitability of industrial operations. Microbial biotechnology is essential to environmental restoration because it uses the metabolic capabilities of microorganisms to degrade pollutants and poisons. Engineered microbes can now lessen pollutants’ detrimental impacts on the environment and promote ecosystem health by targeting them and turning them into harmless byproducts (Ledford and Callaway, 2020). The pharmaceutical industry greatly benefits from microbial biotechnology, particularly in the production of vaccines, antibiotics, and therapeutic proteins. Yeast and *Escherichia coli* are examples of microbial hosts that have been engineered to manufacture complex proteins with high yield and purity, which reduces production costs and expedites the creation of new medications. Furthermore, advancements in fermentation technology have enabled the large-scale production of biopharmaceuticals, met global demand, and ensured product efficacy and safety (Jinek et al., 2012). In response to the increasing need for sustainable energy sources, microorganisms, such as bacteria and algae can promote energy security, offering greener substitutes for fossil fuels. Microbial enzymes have gained recognition globally for their widespread uses in various sectors of industries like food, agriculture, chemicals, medicine, and energy (Choi et al., 2015). Industrial enzymes derived from microbes have continued to change many industrial processes. Protein engineering and enzyme immobilisation have increased their stability, specificity, and efficiency in a variety of applications, including food processing and textile manufacturing. Microbial enzymes offer sustainable alternatives to chemical catalysts by promoting green chemistry practises and reducing their adverse environmental consequences (Charpentier and Doudna, 2013).

### 2.3 Microbial engineering for environmental sustainability

**Figure 4:**
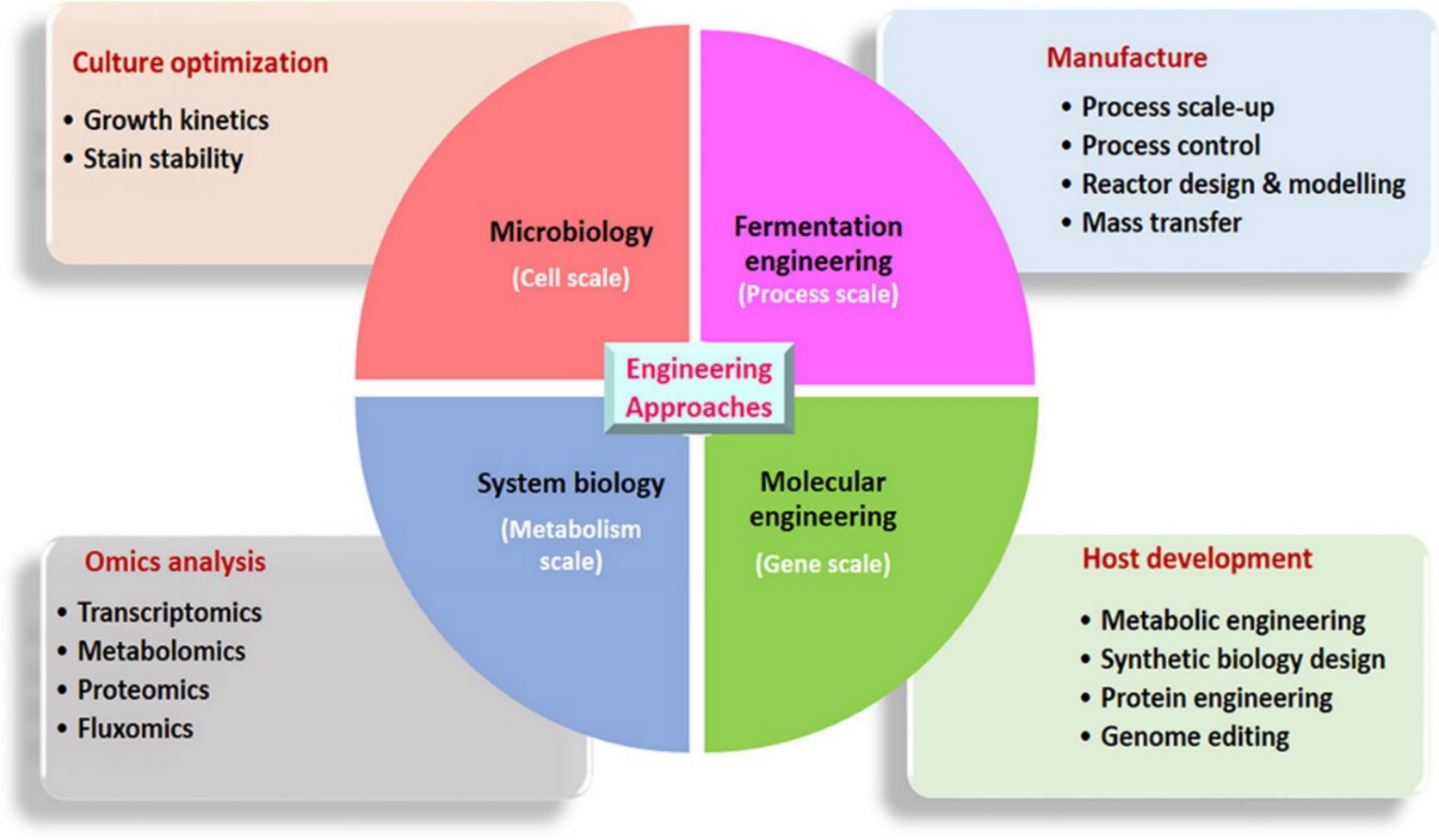
Engineering approaches for enhanced microbial biosynthesis of biofuels (Joshi et al., 2022). Source: https://www.tandfonline.com/action/showCopyRight?scroll=top&doi=10.1080%2F21655979.2022.2051856.

The naturally occurring microorganisms have continually shown metabolic limitations (Cunningham, 2024), implying that engineered microorganisms may hold promise for environmental management and ensure sustainability. On the other hand, microorganisms can develop survival mechanisms to adapt and thrive in diverse environments (Montaño López et al., 2022), and owing to their cooperative behaviour, efficient microbial processes can be achieved using mixed microbial communities (Verstraete et al., 2022). These phenomena can be exploited by scientists to provide numerous environmental benefits. Nonetheless, microbial metabolic limitations can be overcome through advanced techniques, such as genetic engineering (Cunningham, 2024) and synbio technologies (Joshi et al., 2022). The latest advancement in synthetic biology and genomics has improved the understanding of adapting microorganisms for functions beyond traditional ones, and they can offer numerous pathways for enhancing the capabilities of microorganisms for environmental pollutant management, allowing targeting specific pollutants for efficient removal (Cunningham, 2024). Genome-editing tools like CRISPR (clustered regularly interspaced short palindromic repeats) improve microbial genome manipulations (Cunningham, 2024; Thakur et al., 2023) by enabling precise genome alterations, and scientists are now empowered to create novel traits with high productivity titres, efficient substrate metabolism, and tolerance to inhibitors (Joshi et al., 2022), offering opportunities for developing sustainable and eco-friendly pathways for revolutionising industrial operations and achieving a sustainable environment. Synthetic biotechnologies can deliver innovative strategies for reducing the environmental impacts of industries (Ezeako et al., 2024), conferring microorganisms with new or improved metabolic capabilities and selective functions, such as efficient biodegradation of toxic compounds and effective utilisation of waste to produce biomaterials (Montaño López et al., 2022). For example, a recent study by Ezeako et al. (2024) showcased that with the aid of synthetic biology techniques, microorganisms can be engineered to degrade various environmental contaminants such as toxic heavy metals, pesticides, hydrocarbons, pharmaceuticals, and plastics. For quick and efficient degradation of plastics, genetically altered microorganisms can deploy high metabolic performance (Cunningham, 2024), fostering ways for their application as a sustainable and viable strategy to reduce plastic waste. Thus, it is possible to address environmental issues and safeguard planetary health by deploying these technologies. Furthermore, genetically engineered microorganisms, such as bacteria, algae, and fungi, were reported to have demonstrated high degradative capacities and fast adaptation for oil spills, camphor, and several hydrocarbons (Pant et al., 2021). Similarly, a recent study presented the use of genetically engineered microalgae for wastewater treatment (Calatrava et al., 2024). Furthermore, a synthetic microbial consortium comprising two *Pseudomonas putida* strains successfully solubilised polyethylene terephthalate hydrolysate, a critical component of plastics (Bao et al., 2023), and the viability of this approach has been attributed to their environmental resilience, metabolic flexibility, and synergistic assimilation of complex pollutants (Renganathan et al., 2025). Microbial biotechnologies not only transform the management of environmental pollution but also mitigate climate change and address resource depletion (Montaño López et al., 2022). Although their success relies on the effective management of the targeted microbial population (Verstraete et al., 2022). Biotechnologically altered microorganisms for converting environmental organic materials to biofuels have been established in current research (Farooq et al., 2025). The numerous engineering strategies for designing and optimising metabolic pathways of microorganisms for improving and scaling biofuel synthesis are illustrated in Figure 4 (Joshi et al., 2022).

**Figure 5:**
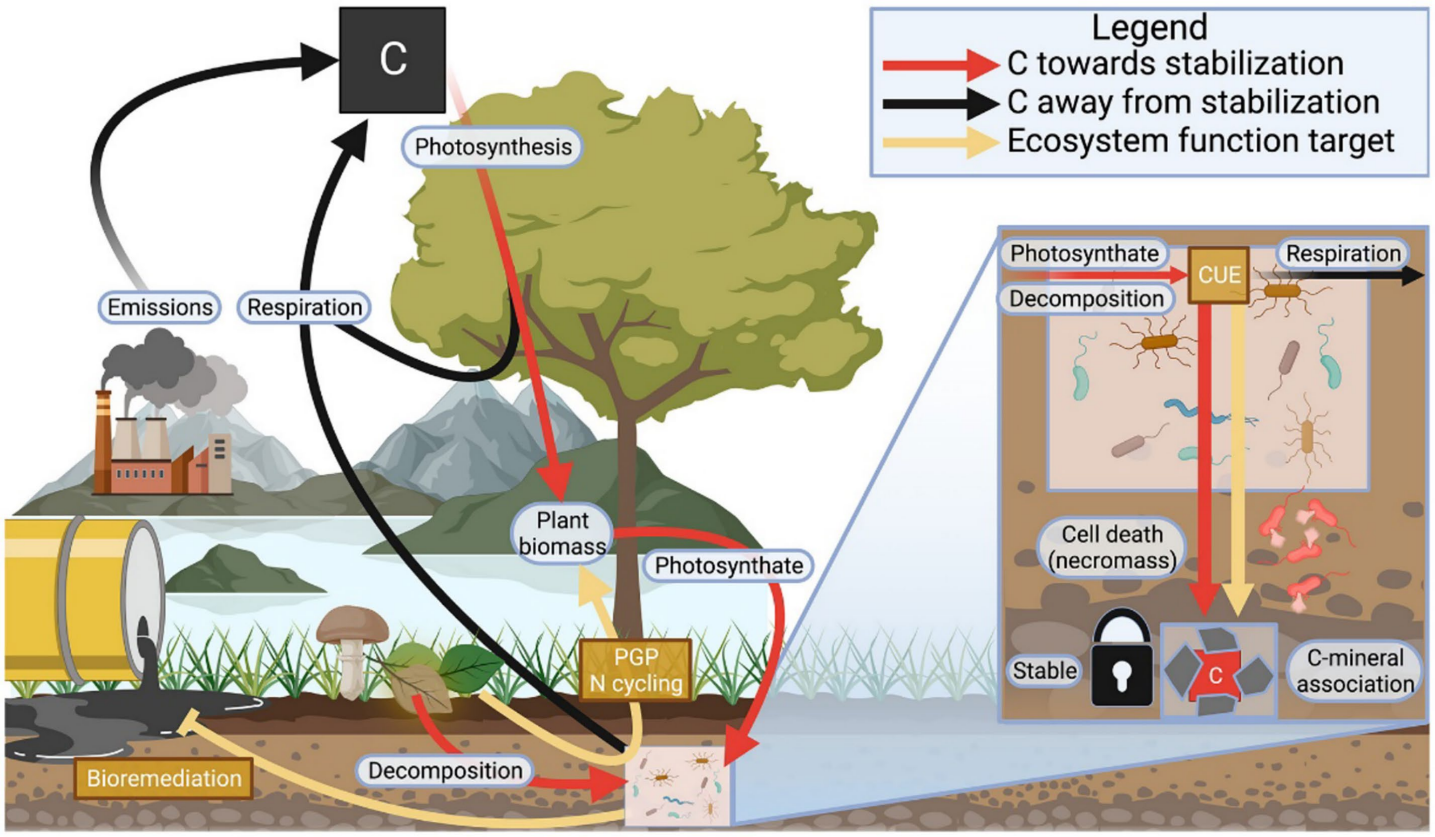
Microbial regulation of carbon dioxide emissions (Silverstein et al., 2023). Source: https://s100.copyright.com/AppDispatchServlet?startPage=2050&pageCount=17&copyright=%C2%A9+2023+The+Authors.+Global+Change+Biology+published+by+John+Wiley+%26+Sons+Ltd.&author=Michael+R.+Silverstein%2C+Daniel+Segr%C3%A8%2C+Jennifer+M.+Bhatnagar&orderBeanReset=true&imprint=John+Wiley+%26+Sons%2C+Ltd&volumeNum=29&issueNum=8&contentID=10.1111%2Fgcb.16609&title=Environmental+microbiome+engineering+for+the+mitigation+of+climate+change&numPages=17&pa=&oa=CC-BY&issn=1354-1013&publisherName=Wiley&publication=GCB&rpt=y&endPage=2066&publicationDate=02%2F08%2F2023.

Genetically engineered photosynthetic bacteria, such as microalgae and Cyanobacteria, were proposed by Calatrava et al. (2024) to enhance industrial carbon capture. Specifically, through a design of experiment approach facilitated by synbio technology, *Synechocystis* sp. has been demonstrated to capture and convert carbon dioxide to bioplastics, providing dual environmental gains (Faulkner et al., 2024). Thus, environmental microbiome engineering that targets carbon regulation by microorganisms for managing carbon dioxide emissions is demonstrated in Figure 5 (Silverstein et al., 2023). These findings point to the endless opportunities engineered microorganisms may bring to achieving environmental sustainability, if explored. Furthermore, the latest advancements in microbial or microbiome engineering, such as the omics (meta-proteomics, meta-transcriptomics, and metabolomics) technologies, have deepened scientists’ understanding on the intricate interactions between organisms and their environment and as such could hold potential for contributing to environmental sustainability (Kaur et al., 2025). Clear details of the taxonomic statuses and functional traits of microorganisms or microbial consortia are being provided by these technologies (Silverstein et al., 2023), creating vital blueprints for precision in engineering microorganisms. Besides these insights have led scientists to new discoveries, such as unknown microorganisms (Shyam et al., 2023), which may improve the current understanding of environmental microbial diversity and their role in maintaining the health and productivity of ecosystems. Metagenomics can be used to analyse environmental genetic materials and has been widely used in bioremediation to identify pollutant degrading microorganisms (Shyam et al., 2023). With the analytical ability possessed by this tool, scientists may be able to develop strategies for overcoming challenges, such as toxicity, with microbial bioremediation technology and ensuring efficiency of the process. In utilising microorganisms for producing various useful products, such as biofuels and biopolymers, understanding the associated molecular mechanisms is critical. Although decoding these molecular mechanisms in biofuel-producing organisms is still at infancy stage (Kumar et al., 2021), omics technologies could contribute to these areas by providing substantial molecular information. Studying these technologies in microalgal system-driven biofuel production, genomic and transcriptomic analysis identified potential genes and regulatory networks associated with fatty acid and triacylglycerol (TAG) synthesis; proteomic studies provided understanding on the enzyme system’s role and impact of post-translational changes; and metabolomic investigations uncovered ways of refining microbial optimisation strategies under different environmental conditions for enhanced biofuel synthesis (Ijoma et al., 2026). Furthermore, application of these technologies in environmental assessment has helped to enhance environmental protection laws (Kesheri et al., 2024), showcasing how biotechnological tools can assist with shaping environmental regulations and policies.

## 3 Limitations and future directions

Microorganisms are cheap resources in the environment, but scaling their metabolic processes for sustainable applications is expensive. For instance, many researchers have made important discoveries related to the biotechnological use of microorganisms for various processes, such as biohydrogen and biopolymer synthesis (Akinsemolu, 2023; Faulkner et al., 2024; de Souza and Gupta, 2024), as well as carbon dioxide sequestration (Srimongkol et al., 2022; Kesheri et al., 2024). However, the cost of scaling these processes has hindered their wide adoption (Akinsemolu, 2023). While substantial funding is also a potential barrier to scaling microalgae technology for carbon dioxide sequestration and assisting with achieving environmental sustainability (Iftikhar, 2023), the technology has failed to effectively trap and secure carbon dioxide, presenting a significant challenge. Utilising extremophilic microorganisms in the bioremediation process presents the challenge of complex adaptation mechanisms, which lead to a decline in microbial growth (Pham et al., 2022), highlighting a critical research gap in the extremophilic microbial bioremediation process, especially under highly unfavourable conditions. Furthermore, a study by Orlando et al. (2023) stresses the need for more studies on new microbial enzymes or engineering enzymes for improved degradation efficiency of polyethylene terephthalate in plastic waste recycling or management, especially in the face of high volumes of polyethylene terephthalate or less pre-treated materials. Because genetically engineered microorganisms arise from alterations of microbial genomes, this technique often affects the genetic diversity of microorganisms (Arora and Fatima, 2024), suggesting a potential ecological ethical concern in terms of direct effect on microbial evolutionary history. Besides, sustaining microbial strains and optimising their metabolic processes for enhanced efficiency require continuous research and development and are usually not economically feasible (Akinsemolu, 2023). Additionally, the introduction of genetic engineering and synthetic biology has provoked safety and ethical questions (Akinsemolu, 2023), which resulted in strong resistance and unacceptance of genetically modified organisms in some regions in addition to weak regulation and policy infrastructures (Arora and Fatima, 2024). Consequently, public acceptance of genetically engineered microorganisms requires clear scientific communication and strong engagement with industries, policymakers, and the public (Akinsemolu et al., 2024). These issues may be a stumbling block for deeper exploration of the potential of these technologies and may consequently prevent their applications in environmental management. Some microorganisms have remained uncharacterised, representing potential opportunities that could be explored (Jansson et al., 2023). The bioremediation activities of microalgae in open wastewater treatment plants can be interrupted by other microorganisms due to the open nature of the plants. These contaminating microorganisms may inhibit the growth of microalgae through competition, thereby impacting the bioremediation process negatively (Srimongkol et al., 2022). Therefore, further investigations into the relationship between microalgae and other microorganisms in open wastewater treatment plants, focusing on their metabolic mechanisms, environmental stressors, and the use of a genome editing approach to improve the bioremedial functions of microalgae and their tolerance traits, should be considered. While advances in microbial biotechnologies limit overdependence on natural resources and combat biowaste management through biofuel synthesis, this practise can encourage the persistence of another environmental issue, impeding the progress of achieving environmental sustainability. For instance, large-scale production of biofuels can lead to deforestation, which can increase greenhouse gas emissions because of the destruction of major carbon storage systems (Arora and Fatima, 2024). Similarly, the application of microalgae for biofuel synthesis requires growth stimulants such as ammonia, whose synthesis is often associated with the release of substantial carbon dioxide, resulting in environmental deterioration (Srimongkol et al., 2022). These issues signal the need for intensive research on both immediate and long-term impacts of microbial technologies on the environment. However, to expand the usage of microalgae technologies in various applications, such as bio-carbon capture, Osorio-Reyes et al. (2023) have recommended further studies towards industrial-scale products recovery techniques, by-products extraction, and optimisation of culture systems, including nutrient concentrations.

## 4 Conclusion

Microbial technologies are pivotal to achieving environmental sustainability. The natural occurrence of microorganisms in every portion of the biosphere and their inherent abilities to multiply quickly and to adapt and thrive in changing environmental conditions have fundamentally made them desirable biological tools for exploitation. Microbiologists provide useful scientific evidence that shows how microorganisms can shape strategies for advancing environmental sustainability. Microorganisms can decontaminate vital environmental compartments such as the soil and water bodies through biodegradation and bioremediation processes. These metabolic processes help to restore, protect, and preserve the environment’s ecological relevance. Some microorganisms have paved their way in the production of biomaterials like biofuels and bioplastics, creating sustainable alternatives to the most important drivers of global environmental issues: fossil fuels and plastics, while preventing over-reliance on the Earth’s natural resources. The recent scientific progress in the use of certain microorganisms, such as the photosynthetic bacteria for biological carbon capture, also show potential microbial applications in climate change mitigation. Advancements in genetic engineering technologies, such as omics tools, synthetic biology, and gene-editing tools, such as CRISPR, can transform microbial metabolic pathways and functions, allowing for designing selective, specific, or precise microbial functions that can be valuable in environmental management. However, despite the beneficial opportunities that microbial technologies present, to realise their full potential for a sustainable environment; issues surrounding their scalability, efficiency, safety, and social acceptance must be addressed.
